# Multi-Attribute Monitoring Method for Process Development of Engineered Antibody for Site-Specific Conjugation

**DOI:** 10.1021/jasms.3c00037

**Published:** 2023-06-02

**Authors:** Alistair R. Hines, Matthew Edgeworth, Paul W. A. Devine, Samuel Shepherd, Nicholas Chatterton, Claire Turner, Kathryn S. Lilley, Xiaoyu Chen, Nicholas J. Bond

**Affiliations:** † Analytical Sciences, Biopharmaceutical Development R&D, AstraZeneca, Cambridge, CB2 0AA, United Kingdom; ‡ School of Life, Health and Chemical Sciences, The Open University, Walton Hall, Milton Keynes, MK7 6AA, United Kingdom; § College of Health, Medicine & Life Sciences, Brunel University London, Middlesex, UB8 3PH, United Kingdom; ∥ Cambridge Centre for Proteomics, Department of Biochemistry, University of Cambridge, Cambridge, CB2 1QR, United Kingdom; ⊥ Analytical Sciences, Biopharmaceutical Development, R&D, AstraZeneca, Gaithersburg, Maryland 20878, United States

**Keywords:** multi-attribute monitoring (MAM), antibody intermediate, antibody-drug conjugate (ADC), site-specific conjugation, biologic manufacturing processes, glycosylation, mass spectrometry (MS)

## Abstract

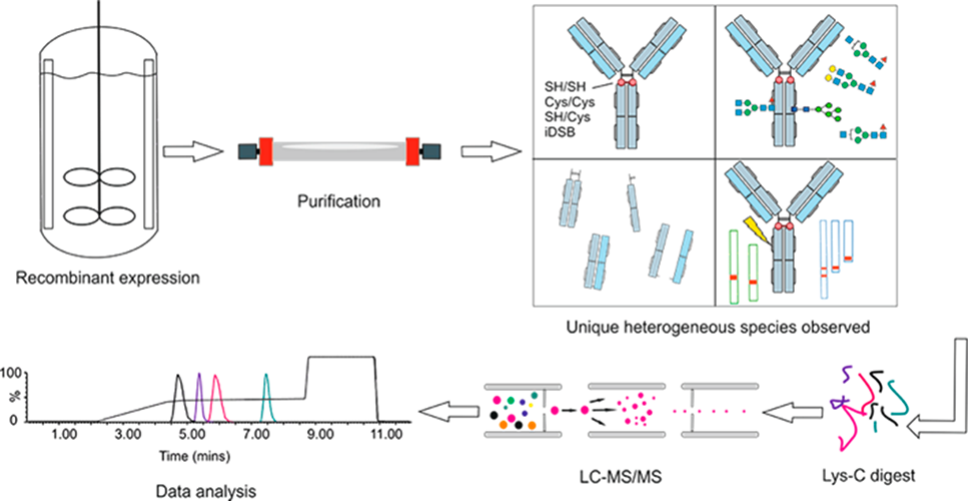

Antibody drug conjugates, a class of biotherapeutic proteins, have been extensively developed in recent years, resulting in new approvals and improved standard of care for cancer patients. Among the numerous strategies of conjugating cytotoxic payloads to monoclonal antibodies, insertion of a cysteine residue achieves a tightly controlled, site-specific drug to antibody ratio. Tailored analytical tools are required to direct the development of processes capable of manufacturing novel antibody scaffolds with the desired product quality. Here, we describe the development of a 12 min, mass-spectrometry-based method capable of monitoring four distinct quality attributes simultaneously: variations in the thiol state of the inserted cysteines, N-linked glycosylation, reduction of interchain disulfide bonds, and polypeptide fragmentation. This method provides new insight into the properties of the antibody intermediate and associated manufacturing processes. Oxidized thiol states are formed within the bioreactor, of which a variant containing an additional disulfide bond was produced and remained relatively constant throughout the fed-batch process; reduced thiol variants were introduced upon harvest. Nearly 20 percent of N-linked glycans contained sialic acid, substantially higher than anticipated for wildtype IgG1. Lastly, previously unreported polypeptide fragmentation sites were identified in the C239i constant domain, and the relationship between fragmentation and glycoform were explored. This work illustrates the utility of applying a high-throughput liquid chromatography–mass spectrometry multi-attribute monitoring method to support the development of engineered antibody scaffolds.

## Introduction

Monoclonal antibodies (mAbs) are an established therapeutic modality, comprising 53.5% of EU and US approvals in between 2018 and 2022, and 51% of new biopharmaceuticals (i.e., not biosimilars) in the same period.^1^ MAbs and mAb-like molecules, whether intended as a drug substance or as an intermediate in the case of antibody drug conjugates, are complex glycoproteins that require mammalian cell lines such as Chinese hamster ovary (CHO) to incorporate the various, necessary, post-translational modifications (PTMs) required for them to function correctly.^1,2^ As such, mAbs are manufactured as an ensemble of closely related product variants which vary in the nature and abundance of their PTMs. Additional modifications can occur upon storage due to the various environmental and chemical stresses experienced. Regardless of their origin, modifications that confer changes to the bioactivity, pharmacokinetics and pharmacodynamics, immunogenicity, and safety of the drug substance are classified as potential critical quality attributes (pCQAs). Establishing the occurrence of pCQAs and controlling their abundance within an appropriate limit, range, or distribution is therefore necessary to ensure the desired product quality of a therapeutic protein is achieved.^3^

These pCQAs include deamidation, isomerization, oxidation, and thiol modifications including scrambling of disulfide bonds, trisulfide bonds, free thiols, cysteinylation, and thio-esters.^4^ Variation in the canonical N-linked glycan located in the Fc region of mAbs contributes toward product heterogeneity, and in some cases can greatly affect antibody drug half-life and efficacy.^5−8^ Depending on their criticality, monitoring these pCQAs through process development and/or upon release has been the mainstay of ensuring consistency and production of a safe and efficacious drug substance.

Recent application of mass spectrometry (MS) technology for measuring pCQAs on chromatographically separated peptides by either high- or low-resolution mass detectors is gaining traction since it presents an opportunity to multiplex the measurement of pCQAs with the potential of enhancing the specificity of release tests. When an MS method is employed to measure more than one attribute within a single analysis, it has been coined a “multi-attribute monitoring” method (MAM).

In a seminal method and paper, Rogers et al. use high resolution Orbitrap mass spectrometry instrumentation and a peptide mapping based sample preparation for monitoring pCQAs across development through to quality control (QC) laboratories.^9,10^ Since this first MAM method was published, there have been several variations of this approach reported, each one seeking to improve the method or apply it to new modalities. Wang et al.^11^ progressed the method by using micro flow liquid chromatography–mass spectrometry (LC-MS) and an ultrafast (5 min) tryptic digest to eliminate the introduction of method-induced modifications (caused by lengthy sample preparation) and monitor antibody oxidation, deamidation, isomerization, glycation, glycosylation modifications, as well as N-terminal pyro-glutamate formation. At-line (i.e., directly from fermentation broth) monitoring of CQAs in cell culture process was achieved by Dong et al.^12^ Reduced mAb (subunit) analysis was performed by Liu et al.^13^ to streamline identification, glycosylation profiling, and ratio determination of coformulated mAbs into a MAM assay using quadrupole-time-of-flight MS. Due to the potential impact of N-glycan heterogeneity on product stability, immunogenicity, and receptor binding and antibody effector function, clearance and half-life, Lanter et al.^14^ used a MAM method to monitor the N-linked glycosylation profile. Choosing to reduce samples taken directly from the bioreactor, this intact mass-based MAM was able to give prompt information about these profiles as measured throughout the cell culture process. A lower resolution approach was taken by Xu et al.,^15^ using a quadrupole Dalton MS (QDa) instrument, replacing several assays in support of cell-culture process development, purification process development, and protein characterization. This method was also qualified for characterizing drug substance and stability samples.^16^ Top-down (TD) and middle-down (MD) MS approaches (analysis of intact protein, and enzymically digested mAb subunits, respectively) have been shown, in an interlaboratory study, to be complementary to each other.^17^ While not coined “MAM”, therapeutic protein integrity, chemical and post-translational modifications, and sequence information were successfully measured. Although sequence coverage using TD/MD methods is commonly less comprehensive than bottom-up approaches (i.e., analysis of proteolytically generated peptides), the experimental procedures are quicker, and carry less risk of preparation induced artifacts.^17^

Several reviews and editorials have been published^18−20^ in recent years which showcase the diversity of applications to MAM methods as well as the methodologies applied.^21^ To our knowledge, no MAM methods have been reported that support the development and manufacture of antibody intermediates engineered for site-specific conjugation.

Antibody drug conjugates (ADCs) are emerging as highly successful cancer treatments, with eight ADC drug products having been approved by the FDA—all for different cancers^22^ with more than 60 ADCs being evaluated in 200 clinical trials.^23,24^ ADCs are constructed by chemically linking a cytotoxic payload to a mAb, with the antibody providing the necessary specificity to target cancerous cells of interest, and the payload having the ability to destroy these cells. For a given target, antibody, and payload combination, the DAR is an important contributor to the therapeutic index and should be tightly controlled. Various conjugation strategies have been employed to achieve this.

The insertion of a cysteine near the hinge region of an antibody IgG1 scaffold (C239i) has been of particular interest, exhibiting favorable properties such as consistent DAR ≈ 2, payload stability over time, decreased FcγR binding, and retaining wildtype half-life by not reducing neonatal Fc receptor (FcRn) affinity.^25^ Recently, we reported changes to the stability, structure, and dynamics resulting from the formation of an unexpected disulfide bond that can occur during antibody manufacture and the subsequent conjugation.^26^ While it does not preclude this format from successful preclinical and clinical development, it highlights that characterizing pCQAs of engineered scaffolds and monitoring these through development is important.

Here, we report the design and utilization of a multiattribute monitoring method for supporting process development of C239i antibody intermediates prior to conjugation. Specifically, we explore a nonreduced LC-MS method targeted to measure thiol states of the inserted cysteine, partial reduction of interchain disulfides, site-specific N-linked glycosylation, and polypeptide fragmentation. The application of this method reveals new insight into the interaction between the manufacturing process and the product quality of this new generation of scaffolds and demonstrates the utility of a high throughput MAM method to direct process development of engineered mAbs.

## Methods

### Materials

Five antibodies (C239i antibody intermediates) were expressed in Chinese Hamster Ovary cells and purified through a multicolumn purification process at AstraZeneca. All intermediates were stored in their formulation buffer at < −70 °C until used. Stressed material was created by incubating at 25 °C for 4 weeks. Material was also chemically altered to have higher than naturally occurring percentages of each of the four thiol state attributes as described in the Results section.

### Multiattribute Peptide Mapping Method

#### NEM Capping, Denaturation, and *Lys-C* Digestion

Solutions used in the NEM capping, denaturation, and *Lys-C* digest were 100 mM sodium phosphate pH 7.0 (Sigma-Aldrich), guanidine hydrochloride (Sigma-Aldrich), sodium chloride (Sigma-Aldrich), dithiothreitol (DTT), N-ethylmaleimide (NEM) (Sigma-Aldrich), EDTA (Merck), and Endopeptidase *Lys-C* (FUJIFILM). No further purification was performed on these reagents.

Free thiols of antibody samples (25 μg of antibody) were capped using 5 μL of 0.5 mg/mL NEM at ambient temperature for 20 min. Samples were then rotary vacuum evaporated for 1 h (or longer to achieve dried pellets). The samples were then reconstituted in 15 μL of a denaturing solution of 8 M guanidine hydrochloride with 5% 2 M sodium chloride and 5% 100 mM sodium phosphate pH 7.0 and incubated at 37 °C for 30 min. The solution was then diluted in 100 mM sodium phosphate_,_ 0.16 mM EDTA pH 7.0, to achieve a guanidine concentration of 2 M in preparation for digest. A 0.5 μg aliquot of *Lys-C* was added to the thiol-capped denatured protein, creating a 1:50 enzyme-to-protein ratio, and the mixture was incubated at 37 °C for 2 h. A further 10 μL (0.5 μg) of *Lys-C* was added and incubated for a final 2 h. Samples were then analyzed or stored at < −70 °C.

#### Reversed Phase LC-MS Method for MAM

Mobile phase A contained 0.02% trifluoroacetic acid (TFA) in water, and Mobile phase B contained 0.02% TFA in 100% acetonitrile. The following LC conditions were used: flow rate 0.15 mL/min, column temperature 55 °C, and autosampler 4 °C. Injections of 10 μL of ∼0.42 mg/mL peptide sample were separated using a UPLC Peptide CSH C18, 130 Å pore size, 1.7 μm bead size, 2.1 mm × 150 mm column (Waters Acquity). The gradient started at 0% B for 2 min, increased to 24% B over 2 min then to 26% B over 0.5 min, then gradually increased to 26% over 3.5 min, stepped up to 80% for 0.1 min and held for 2 min. The gradient was dropped back to 100% A for 1 min to equilibrate for the next injection. Total run time per sample was 12 min.

After separation, the *Lys-C* digested peptides were analyzed by a triple quadrupole mass spectrometer (Waters Xevo TQS).

#### Data Processing

The targeted MS data was exported from the TargetLynx (Waters) software into a Microsoft Excel spreadsheet where data was reported, sample by sample, for each transition. Each transition was designated as “compound” by the TargetLynx software. A Matlab (Mathworks) script was implemented to extract information (such as relative percentages) from samples and pivot tables in a fashion that allows easier post processing in Microsoft Excel.

#### Reducing Capillary Gel Electrophoresis (Sciex PA800 Plus)

The reducing capillary gel electrophoresis was performed by first diluting the antibody intermediate samples to 0.5 mg/mL in a 100 mM sodium phosphate buffer pH 6.0 containing 4% sodium dodecyl sulfate (SDS). Beta-mercaptoethanol was added for a final concentration of 5%, and the mixture was heated for 5 min at 65 °C. The denatured and reduced samples were analyzed on a Sciex PA800 plus CE system.

#### Nonreducing Microfluidic Gel Electrophoresis (Agilent BioAnalyzer)

The nonreducing microfluidic gel electrophoresis was performed by first diluting the antibody intermediate to 4 mg/mL in 1X phosphate buffered saline. NEM was added to the kit sample buffer to produce a 60 mM NEM alkylating sample buffer, which was mixed at a 1:1 ratio with the 4 mg/mL sample. The mixture was heated at 80 °C for 1 min, and 6 μL of this solution was added to 84 μL of water prior to analysis on an Agilent 2100 Bioanalyzer system.

#### HILIC 2-AB oligosaccharide profiling

HILIC oligosaccharide profiling of the C239i samples was conducted through UPLC analysis. Antibody intermediate (100 mg) was digested overnight with PNGaseF (V4831, Promega) at 37 °C, and subsequently labeled with 2-aminobenzamide (PN 654213, Sigma-Aldrich) for 30 min at 37 °C. The labeled oligosaccharides were extracted using GlykoClean SPE Cartridges (GC210, Prozyme), which were then injected onto a Waters ACQUITY UPLC fitted with a Glycoprotein Amide Column (186007963, Waters) and detected by fluoresce detection.

#### Reduced Tryptic Peptide Mapping

Reduced tryptic peptide mapping was performed by first diluting 50 μg of antibody intermediate to 10 mg/mL. Samples were denatured and reduced at 37 °C for 30 min in 20 μL of 6.3 M urea, 1 M guanadine HCl, 100 mM Tris pH 8.0, and 5 mM dithiothreitol (DTT, Thermo Scientific) mixture. Then, samples were alkylated with 15 mM iodoacetamide (IAM, Thermo Scientific) for 30 min in the dark at room temperature. Subsequently, the reaction was diluted with 3 volumes of 100 mM Tris pH 7.5 to allow for trypsin digestion. Trypsin (V5280, Promega) was added at 1:12.5 protease: protein ratio and incubated at 37 °C for 3–4 h. The reaction was quenched by adding 5 μL of 10% TFA (T6508, Sigma-Aldrich). The digests were analyzed by LC-MS using a Waters ACQUITY UPLC system equipped with a Waters ACQUITY BEH C18 column (1.7 mm, 2.1 × 150 mm), mobile phase A (0.02% TFA in HPLC water), mobile phase B (0.02% TFA in acetonitrile) over a 90 min gradient, and a Synapt G2 mass spectrometer (Waters).

#### Intact and Reduced LC-MS

Samples for intact LC-MS analysis were diluted to 1 mg/mL using 50 mM Tris pH 8.0 and diluted in a 1:1 ratio with PNGase F (Promega) and incubated at 37 °C for 16–20 h to deglycosylate. Samples for reduced LC-MS were diluted to 1 mg/mL in 50 mM Tris pH 8.0 and reduced by incubating for 30 min at 37 °C with addition of 2 μL DTT solution.

Samples were analyzed by LC-MS using a Waters ACQUITY UPLC system equipped with a Waters ACQUITY BEH C4 column (1.7 μm, 2.1 × 50 mm), mobile phase A (0.01% TFA, 0.1% FA in water), mobile phase B (0.01% TFA, 0.1% FA in acetonitrile), and a Synapt G2 mass spectrometer (Waters).

## Results

### Thiol States of C239i

**Figure 1:**
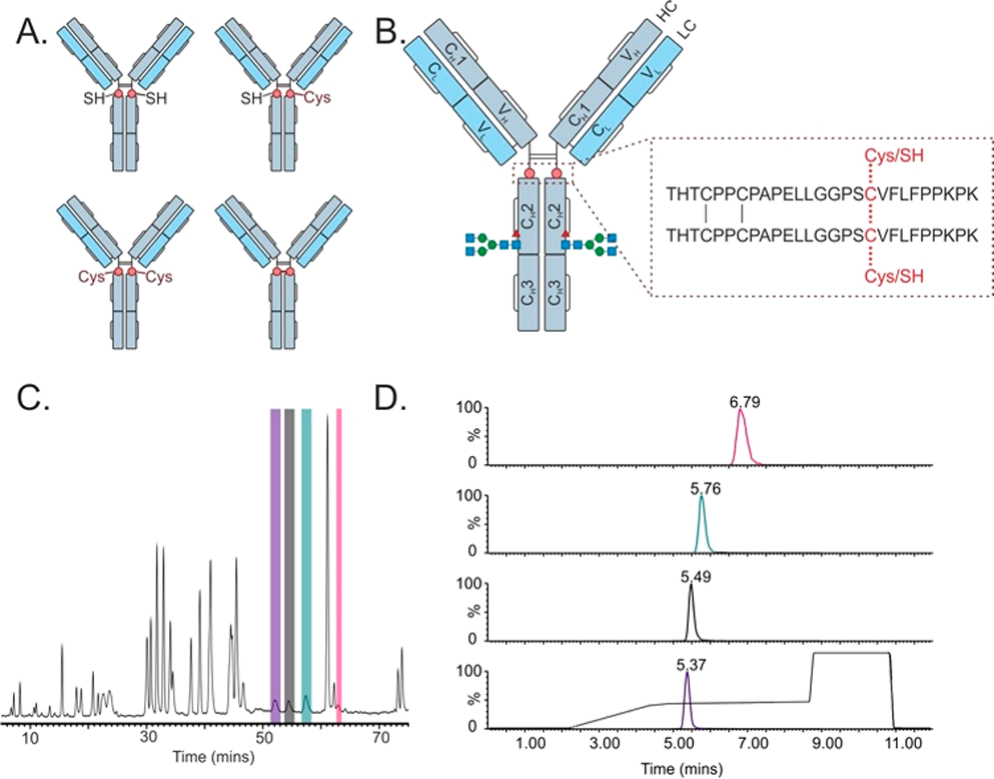
Detail of thiol states, analysis by MAM method, and comparison to NRPM. A. Schematic of the antibody intermediate containing the inserted cysteine at position 239 (C239i), in four forms: free thiol (2xSH), one cysteinylation and one free thiol (1xSH/1xCys), forming an additional disulfide bond (iDSB), and doubly cysteinylated (2xCys). B. Hinge peptide sequence illustrating different thiol states. C. Nonreduced peptide mapping (NRPM) data of C239i antibody intermediate which exhibited all 4 thiol states: 2xSH (pink), 1xSH/1xCys (green), iDSB (black), 2xCys (purple). D. 12 min LC gradient highlighting the chromatographic separation of the 4 thiol states monitored within the assay.

Initial characterization across four antibody intermediates revealed that the inserted cysteine at position C239 could adopt several, distinct thiol states during manufacture: free thiol (2xSH), cysteinylated (1x/2x Cys), and an additional disulfide bond between both C239i residues (iDSB) (Figure 1A, C). These findings correlate with those of Orozco et al.^26^ and Cao et al.^27^ (2xSH and iDSB variants discussed only). Orozco et al.^26^ used high resolution tandem mass spectrometry and the ensuing unique peptide fragmentation patterns to confirm that the additional disulfide bond was configured similarly to the canonical mAb interchain disulfide bridge—a heavy–heavy link at equivalent sites (C239) of each chain.

While the mass difference of cysteinylated forms can be measured by protein-level mass spectrometry, the free thiol and iDSB forms that differ by 2 Da (13 ppm) could not be adequately resolved such that mixed populations could only be accurately measured following digestion under nonreducing conditions. The free thiol and iDSB forms (of the peptide containing C239i) were readily distinguishable once NEM-capped and digested; however, the free thiol (capped), singly cysteinylated, and doubly cysteinylated forms produced precursors of similar *m*/*z* (1479.7, 1478.2, and 1476.7, respectively) and needed chromatographic separation to avoid codetection. Transitions were configured to measure each thiol state: unique *m*/*z* filters that were specific for each thiol state precursor were applied to the first quadrupole (Table 1). A second *m*/*z* filter was applied to the third quadrupole which selected the most abundant (and common) fragment ion, y5, to maximize sensitivity and facilitate relative quantitation between thiol states. Cross-talk between transitions was avoided by optimizing the reversed phase gradient to provide chromatographic separation between thiol states of similar *m*/*z* (Figure 1D). To quantify the proportion of any given thiol state, the detected signal of each state was divided by the cumulative signal (S) across all states (eq 1).
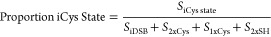
1

**Table 1:** Transitions for Attributes Monitored and the Corresponding Peptides and Modified Variants Thereof^a^

Attribute	Compound [z]	RT window (minutes)	*m*/*z* Q1	*m*/*z* Q3 (frag. ion)
Fragmentation	H310–H317 [2+] (Frag 1) HQDWLNGK	4.00–4.80	499.20	204.12 (y2)
	H310–H317 [2+] (Frag 1) HQDWLNGK	4.00–4.80	499.20	318.16 (y3)
	H278–H288 [2+] (Frag 2) YVDGVEVHNAK	3.80–4.40	615.80	469.20 (y4)
	H278–H288 [2+] (Frag 2) YVDGVEVHNAK	3.80–4.40	615.80	968.40 (y9)
	H307–H317 [2+] (Frag 3) TVLHQDWLNGK	4.40–4.80	655.82	204.12 (y2)
	H307–H317 [2+] (Frag 3) TVLHQDWLNGK	4.40–4.80	655.82	318.16 (y3)
	H277–H288 [2+] (Frag 4) WYVDGVEVHNAK	4.30–5.10	708.80	469.20 (y4)
	H277–H288 [2+] (Frag 4) WYVDGVEVHNAK	4.30–5.10	708.80	968.40 (y9)
	H275–H288 [2+] (wildtype) FNWYVDGVEVHNAK	4.60–5.30	839.35	469.20 (y4)
	H275–H288 [2+] (wildtype) FNWYVDGVEVHNAK	4.60–5.30	839.35	968.40 (y9)
	Standard IgG H393–H409 [2+] TTPPVLDSDGSFFLYSK	5.10–5.60	937.46	397.21 (y3)
	Standard IgG H393–H409 [2+] TTPPVLDSDGSFFLYSK	5.10–5.60	937.46	836.43 (y15)
Partial Reduction	Lambda L209–216; H218–221 [2+] TVAPTECS-SCDK	3.60–4.40	628.76	484.20 (y5-H20)
	Lambda L209–216; H218–221 [2+] TVAPTECS-SCDK	3.60–4.40	628.76	658.20 (y2)
	Kappa L208–214; H223–226 [2+] SNFRGEC-SCDK	3.50–4.50	631.25	562.30 (b5)
	Lambda L209–216 [1+] TVAPTECS	4.20–4.75	932.45	661.30 (y5)
	Lambda L209–216 [1+] TVAPTECS	4.20–4.75	932.45	732.40 (y6)
	Kappa L208–214 [1+] SNFRGEC	4.00–4.60	937.38	505.30 (b4)
	Kappa L208–214 [1+] SNFRGEC	4.00–4.60	937.38	562.30 (b5)
	H222–H248 [3+] (reduced) THTCPPCPAPELLGGPSCVFLFPPKPK	5.50–7.50	1070.58	1321.80 (y23)
	H222–H248 [3+] (reduced) THTCPPCPAPELLGGPSCVFLFPPKPK	5.50–7.50	1070.58	1435.85 (y24)
Glycosylation^b^	Man5 [4+] H289–H317	4.80–5.50	1170.34	138.00
	G0 [4+] H289–H317	4.80–5.50	1191.07	138.00
	G1F-GN [4+] H289–H317	4.80–5.50	1216.83	138.00
	G0F [4+] H289–H317	4.80–5.50	1227.59	138.00
	G1F [4+] H289–H317	4.80–5.50	1268.1	138.00
	G1F-GN+NAc [4+] H289–H317	4.80–5.50	1291.4	138.00
	G2F [4+] H289–H317	4.80–5.50	1308.6	138.00
	G1FS [4+] H289–H317	4.80–5.50	1340.8	138.00
	G2FB [4+] H289–H317	4.80–5.50	1358.88	138.00
	G2FS [4+] H289–H317	4.80–5.50	1381.60	138.00
	G2FS2 [4+] H289–H317	4.80–5.50	1454.41	138.00
Thiol state^c^	iDSB [4+] H222–H248	5.10–7.50	1416.5	566.30 (y5)
	2xCys [4+] H222–H248	5.10–7.50	1476.7	566.30 (y5)
	1xSH + 1 Cys [4+] H222–H248	5.10–7.50	1478.2	566.30 (y5)
	2xSH [4+] H222–H248	5.10–7.50	1479.7	566.30 (y5)

a Detail of peptide location is given using protein name (H or L for heavy and light chain respectively) and residue location starting from the N-terminus, e.g., H1–H12 is a peptide that comprises residues 1–12 of the heavy chain.

b Glycopeptide: TKPREEQYN[glycoform]STRVVSVLTVIHQDWLNGK.

c Masses for 1x and 2x SH are capped and therefore include mass for NEM.

**Figure 2:**
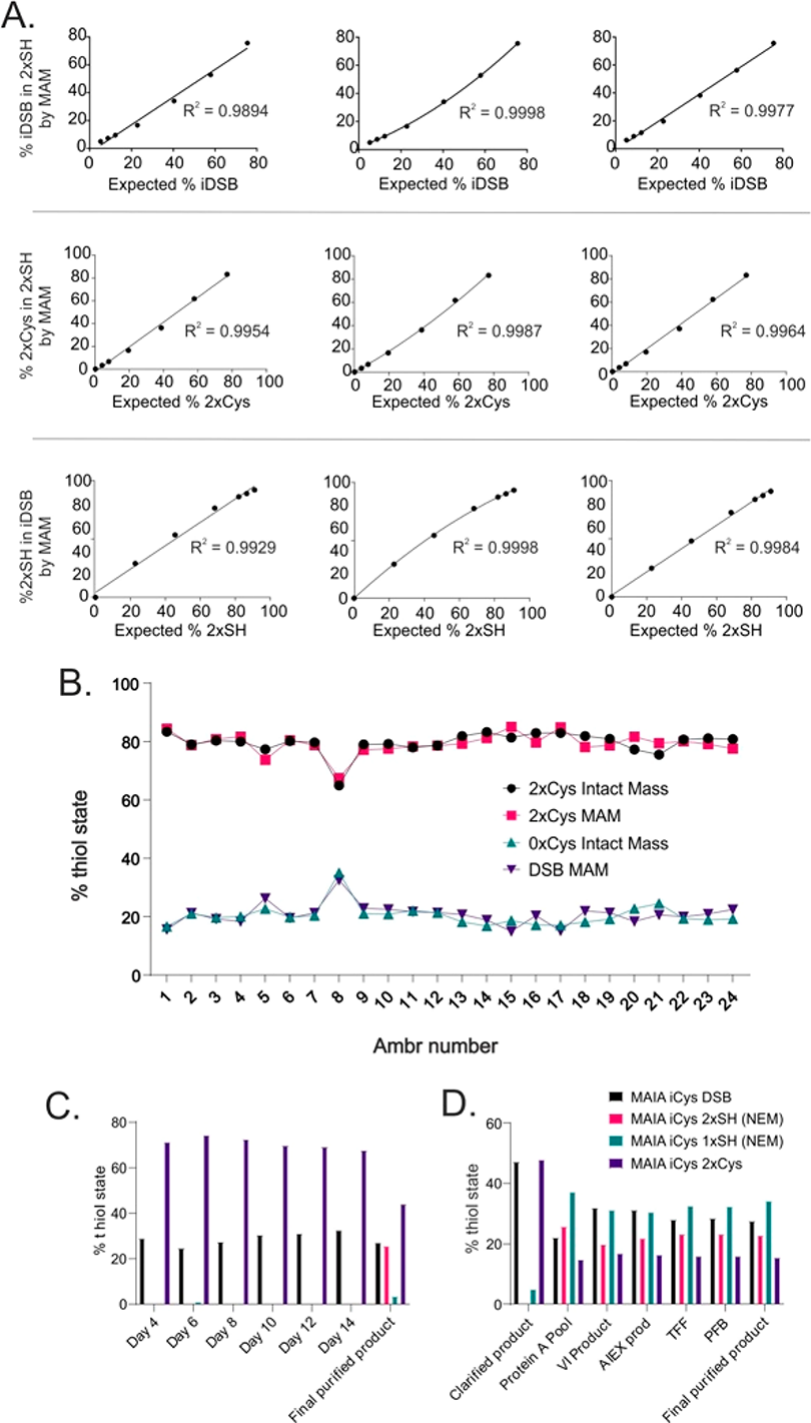
Thiol state ionization bias correction and MAM analysis of process development samples. A. Calibration curves depicting expected thiol state (expected amount from spiking of one near-homogeneous thiol variant into another) compared to amount measured by the MAM method. Each row shows a different blend of thiol variants, with each column showing a modification to the data analysis—the leftmost column showing a linear regression, the center column a nonlinear second-order regression, and the rightmost column having had a correction factor applied which corrects for ionization bias between 2xSH and all other forms. B. Comparison of measurement of thiol state by intact mass LC-MS and the MAM method in 24 different stable cell lines expressing the same antibody intermediate. C and D. Analyses of thiol states utilizing the MAM method for automated miniature bioreactor (Ambr) time points, and stages of the purification process, respectively—purification stages are in order, left to right. *Clarified product: sample taken after clarification and analyzed; Protein A pool: sample taken after Protein A purification; VI product: sample taken after viral inactivation through holding material at low pH and then neutralizing before next purification step; anion exchange product: sample taken after anion exchange chromatography step; TFF: sample taken after material buffer exchanged to final buffer and concentration utilizing tangential flow filtration; PFB: sample taken is preformulated bulk.

To explore if this signal was linear, calibration curves were generated where material enriched for either iDSB or doubly cysteinylated (2xCys) were spiked into doubly free thiol material (2xSH). The resulting linear regression achieved a least-squares correlation (R^2^) of 0.99 between expected and observed thiol state proportions. Despite the good linear correlation, closer fit could be achieved by using a second-order exponential demonstrating minor differences in the ionization propensity of cysteinylated and iDSB states with respect to the free thiol form, and the subtle underestimation of these modified states (Figure 2A). Application of a correction factor to the detected signal of cysteinylated and iDSB forms with respect to 2xSH corrected for this resulting in an R^2^ of > 0.995.

The MAM method was deployed to monitor the C239i thiol state during process development. Analysis of 24 clones cultured at a 15 mL scale in automated miniature bioreactors (Ambr) revealed that oxidized thiol states were exclusively adopted, and that the proportion of iDSB was present at approximately 20% and largely independent of clone (Figure 2B). The abundance of cysteinylation agreed with measurements made by intact mass analysis, post-deglycosylation. Intact mass analysis, however, cannot readily distinguish the free thiol form from the iDSB form. When applied to monitor thiol state during the fed batch culture, MAM analysis revealed that the amount of iDSB and cysteinylated states remained constant throughout the 14-day bioprocess (Figure 2C). However, the final antibody intermediate (AI) revealed the presence of reduced thiol states (1xSH, 2xSH), suggesting these were introduced during downstream processing (Figure 2C). Investigating further, MAM analysis revealed the introduction of reduced thiol states during harvest/protein A purification, after which the relative proportions of thiol states remained relatively unaffected by each subsequent purification step (Figure 2D). Further characterization revealed that elevated temperature, oxidizing environment, and absence of glycan all accelerate the formation of iDSB (Figure S5).

### Glycosylation

**Figure 3:**
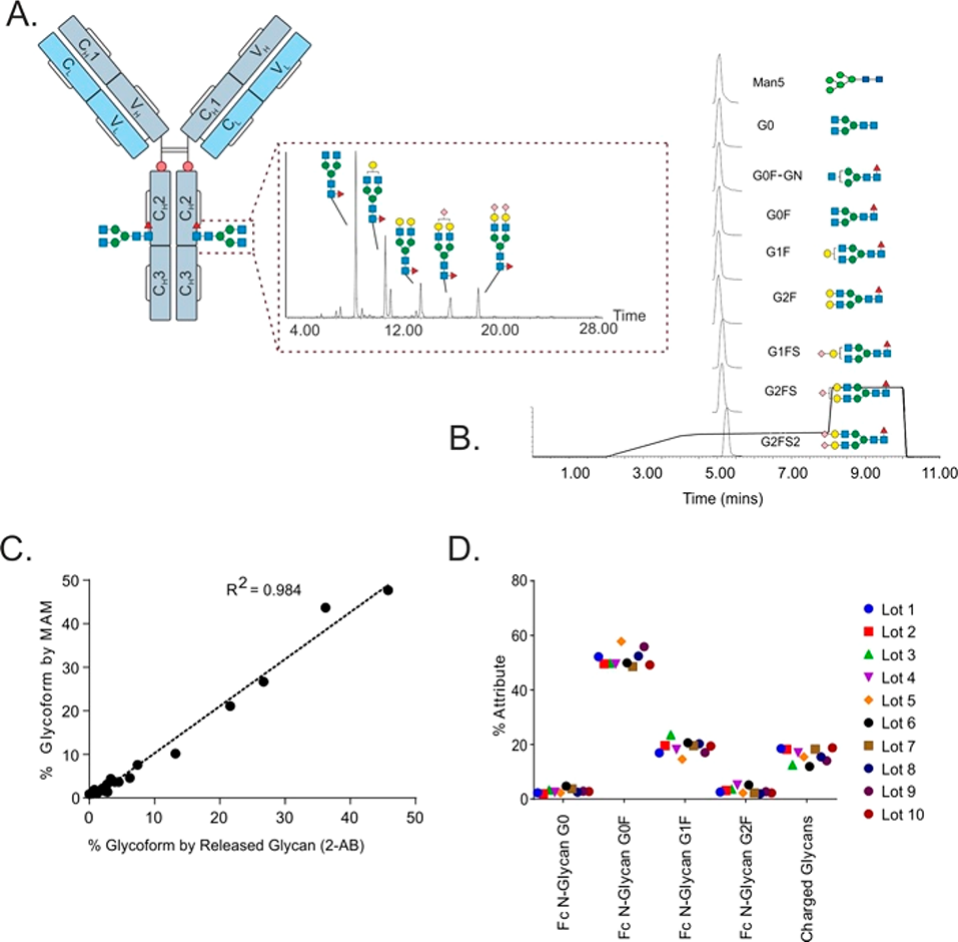
Comparison of glycosylation observed by MAM & 2-AB and MAM analysis of process development samples. A. Schematic illustrating N-linked glycosylation site with examples of glycoforms observed and a typical profile as observed by 2-AB method. B. Labeled multiple reaction monitoring (MRM) transitions and associated structure for each of the glycoforms as measured by the MAM method with the LC gradient overlaid. C. Comparison of MAM measurement to 2-AB (traditional method using fluorescent detection of labeled and separated glycans following enzymatic release from the antibody intermediate). D. Demonstration of Fc N-linked glycoform consistency across ten lots of C239i mAb4.

Site-specific N-linked glycosylation of IgG antibodies at position Asn297 is an important, conserved post-translation modification that occurs in the endoplasmic reticulum and Golgi subcellular compartments generating complex-, hybrid- or high mannose-type glycoforms^7^ (Figure 3A illustrates the location of this PTM). Despite this potential for substantial heterogeneity, the Fc of IgG1 therapeutic proteins reported in the literature are predominantly decorated with neutral glycans of the complex, biantennary type, such as G0f, G1f, G2f with low (< 10%^28^) or undetectable/negligible^7^ levels of sialylated species such as G1fS, G2fS, G2fS2. Interestingly, characterization of four C239i antibody intermediates during early development revealed that the proportion of charged, sialylated glycans ranged between 10 and 20% as determined by hydrophilic interaction chromatographic (HILIC) separation and fluorescence detection of released, 2-aminobenzamide (2-AB)-labeled glycans (data not shown), with the MAM method corroborating levels of sialylated glycoforms.

Sialylated glycans attached to CHO-expressed mAbs have been shown to interfere with FcγRIIIa binding and reduce antibody-dependent cellular cytotoxicity (ADCC),^6^ have anti-inflammatory activity,^29^ and have potential to be immunogenic in humans;^7^ mice studies have also indicated that a decreased IgG half-life was observed upon sialic acid removal.^7^ These reasons, in addition to the importance of controlling charged variants during manufacture, make site-specific glycosylation, especially sialylated species, an important attribute to monitor.

To measure the expected glycoforms in a high throughput and multiplexed manner, transitions were configured for each. Mass-to-charge filters in the first quadrupole were selected to be specific for the various glycoforms of the predominant glycopeptide released by *LysC* digestion: TKPREEQYN[glycoform]STRVVSVLTVIHQDWLNGK (Figure 3B contains details of the glycoforms measured). Oxonium fragment (b) ions proved to give enhanced sensitivity over polypeptide fragment (y) ions across a range of collision energies. Since oxonium ions offer little specificity and can suffer interference from some peptide fragments,^30^ each transition was confirmed using high resolution mass spectrometry (data not shown) prior to being taken forward for further optimization. The response for three oxonium ions, namely 138 *m*/*z* (GlcNAc minus −CH–2H_2_O), 204 *m*/*z* (GlcNAc), and 366 *m*/*z* (GlcNAc-Man or GlcNAc-Gal) were explored as a function of cone voltage (V), source offset (V) and collision energy by a design of experiments (DoE) approach. Whilst the model revealed (with significance, *p* < 0.01) that the response of oxonium ions with collision energy or cone voltage was not linear, the influence of these parameters on the sensitivity of the assay was modest. Instead, the choice of oxonium ion itself had the largest bearing on the sensitivity of detecting the glycopeptide and was independent of glycoform, with the most sensitive oxonium ion being GlcNAc minus −CH–2H_2_O (138 *m*/*z*) (Figure S1). These data-supported glycopeptide detection is most sensitive when operating within a parameter space of 40–50 V for cone voltage, 50–60 V for source offset, and 70–80 V for collision voltage.

Glycopeptides are prone to in-source fragmentation, where larger, particularly sialylated, glycoforms decompose to smaller glycoforms and by doing so introduce ambiguity into their quantitation. To address this, the results of the DoE were scrutinized to find conditions that minimize in-source fragmentation but revealed that none of the parameters evaluated significantly contributed toward the in-source fragmentation observed. Further experimentation identified that in-source fragmentation was instead reduced by decreasing the capillary voltage, nebulizing flow rate and source temperature. A second DoE was conducted which confirmed that desolvation temperature (*p* = 0.0001) and desolvation gas flow (*p* = 0.0019) and to a lesser extent capillary voltage (*p* = 0.006) were all significantly correlated with in-source fragmentation (Figure S2). An assessment on the response of the 138 *m*/*z* oxonium ion determined that negligible in-source fragmentation could be achieved without compromising on sensitivity by operating within a source parameter space of capillary voltage at 3 kV, nebulizing flow rate of 400 L/H, and source temperature of 350–450 °C.

To accurately quantify the proportion of glycoforms present in a sample, the differential ionization and fragmentation of each glycoform also needed to be accounted for. Here, we calculated conversion factors using a subset of samples comprising different lots of four C239i mAbs where the glycoforms had been measured by both MAM and the 2-AB method. To validate this approach, a second subset of samples were measured by MAM and 2-AB, and the MAM data corrected for ionization and fragmentation using the predetermined correction factors. This data shows a correlation (R^2^ > 0.98) between the quantitation of nine glycoforms detected by released glycan and the MAM method (Figure 3C). Once comparability to the orthogonal (2-AB) method was demonstrated, the MAM method was routinely used to rapidly characterize these site-specific N-glycans throughout the antibody manufacturing process. For C239i mAb4, glycans were evaluated across 10 lots of antibody intermediate (Figure 3D), revealing a consistently higher level of sialylated glycoforms than expected for a wildtype IgG1 (Figure S3).

### Partial Reduction

**Figure 4:**
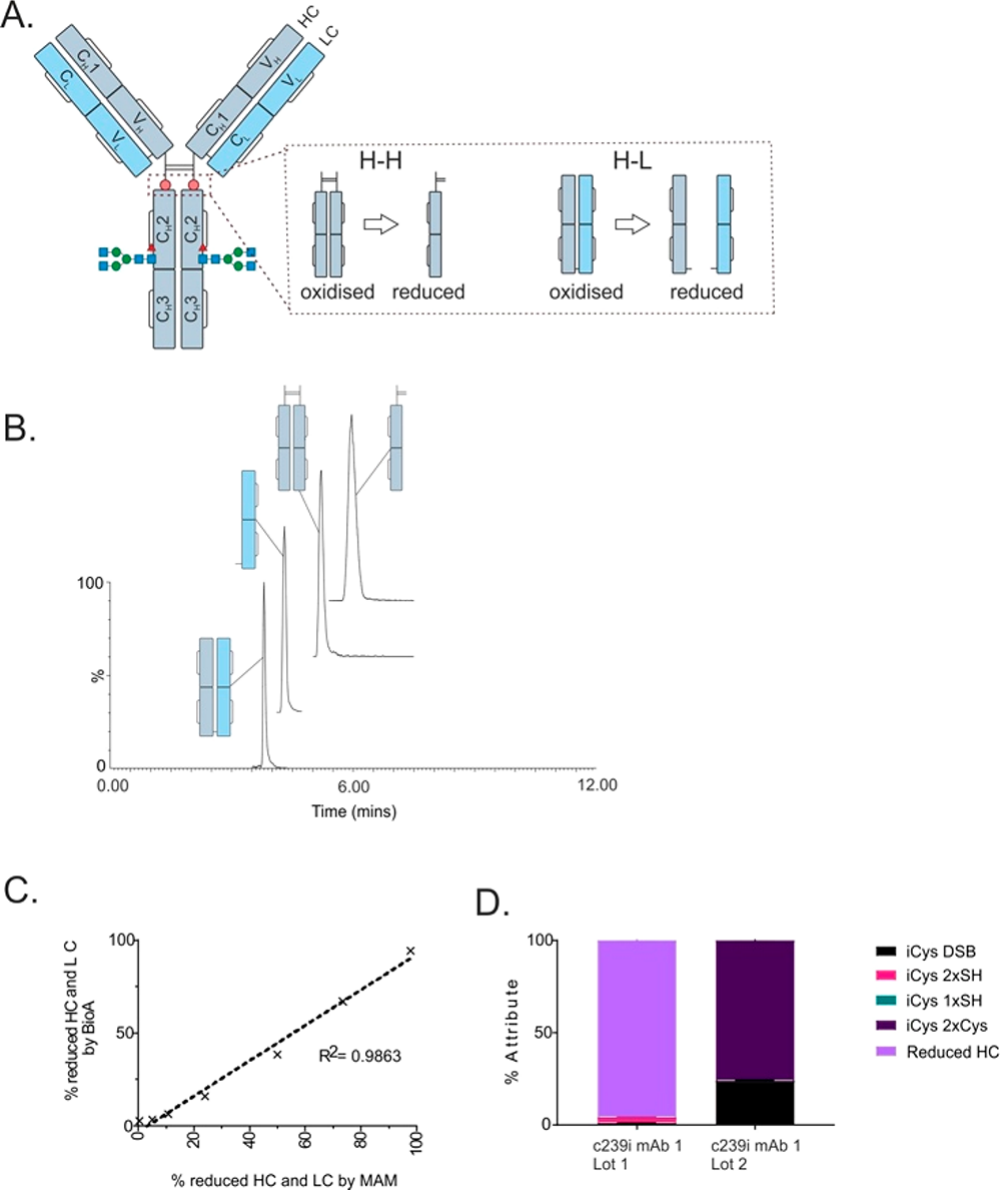
Disulfide bond integrity analysis, comparison to BioA, and MAM analysis of process development samples. A. Pictorial representation of oxidized and reduced peptides monitored. Shown are the heavy–heavy and heavy–light disulfide bonds when formed (oxidized) and broken (reduced). B. Labeled MRM transitions to assess disulfide bond integrity and associated structure for each of the peptides measured by the MAM method, including oxidized and reduced forms of antibody heavy chain, kappa, and lambda light chains. C. Comparison of MAM measurement to traditional measurement (nonreducing microfluidic gel electrophoresis (Agilent BioA)). D. Comparison of two large-scale lots of the same antibody intermediate, C239i mAb 1, one observing high levels of interchain disulfide bond reduction.

Therapeutic antibodies adopt a quaternary structure composed of two heavy and two light chains stabilized by disulfide bonds. For the C239i antibody intermediate, a single disulfide bond is expected between heavy and light chains, with two disulfide bonds between the heavy chains (Figure 4A). Interchain and intrachain disulfide bonds are responsible for stabilizing the protein against unfolding and disassociation^31,32^ but have been previously reported to undergo partial reduction during the manufacturing process.^33^ While the integrity of the disulfide bonds in not necessarily a critical quality attribute for a mAb intermediate, examples have been reported where these cysteines have been exploited for conjugation strategies and have shown that a homogeneous ADC created by partially reducing disulfides prior to conjugation showed fewer adverse effects than the wild type upon evaluation of the safety profile, in animal studies.^34^ It is nevertheless important to ensure the consistency of all quality attributes given the expectations of regulators for releasing the mAb intermediate as a drug substance.

To quantify the extent of partial reduction by MAM, transitions specific to the correctly assembled chains and to the products of disulfide bond reduction were designed (Figure 4B). Since human antibodies contain either kappa or lambda light chains which exhibit different C-terminal sequences, two sets of transitions were necessary to establish a generic assay for all C239i intermediates, regardless of light chain isotype. Differences in the detected signal for equimolar amounts of reduced or disulfide-bonded forms is expected due to differential ionization of fragmentation patterns of peptides of each. To correct for these, a sample which was fully reduced and NEM-capped was mixed in equal proportions with the starting material (nonreduced), and correction factors determined which could be used to normalize the signals detected. Next, to evaluate if the detection of partial reduction by MAM was linear, calibration curves were generated by spiking fully reduced and NEM-capped samples back into the starting material at known concentrations (Figure S4). After correction, the proportion of partial reduction at each bond could be reported and when summed and compared to nonreducing capillary electrophoresis demonstrated a good correlation (Figure 4C). MAM analysis was used to monitor partial reduction. To quantify the proportion of partial reduction, the detected signal of the reduced heavy chain was divided by the cumulative signal of all iCys states.
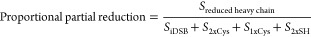
2

In one instance, unusually high levels of reduced interchain disulfide bonds were observed during large-scale manufacture . A subsequent lot was manufactured where the expected disulfide bond integrity was observed (Figure 4D).

### Fragmentation

**Figure 5:**
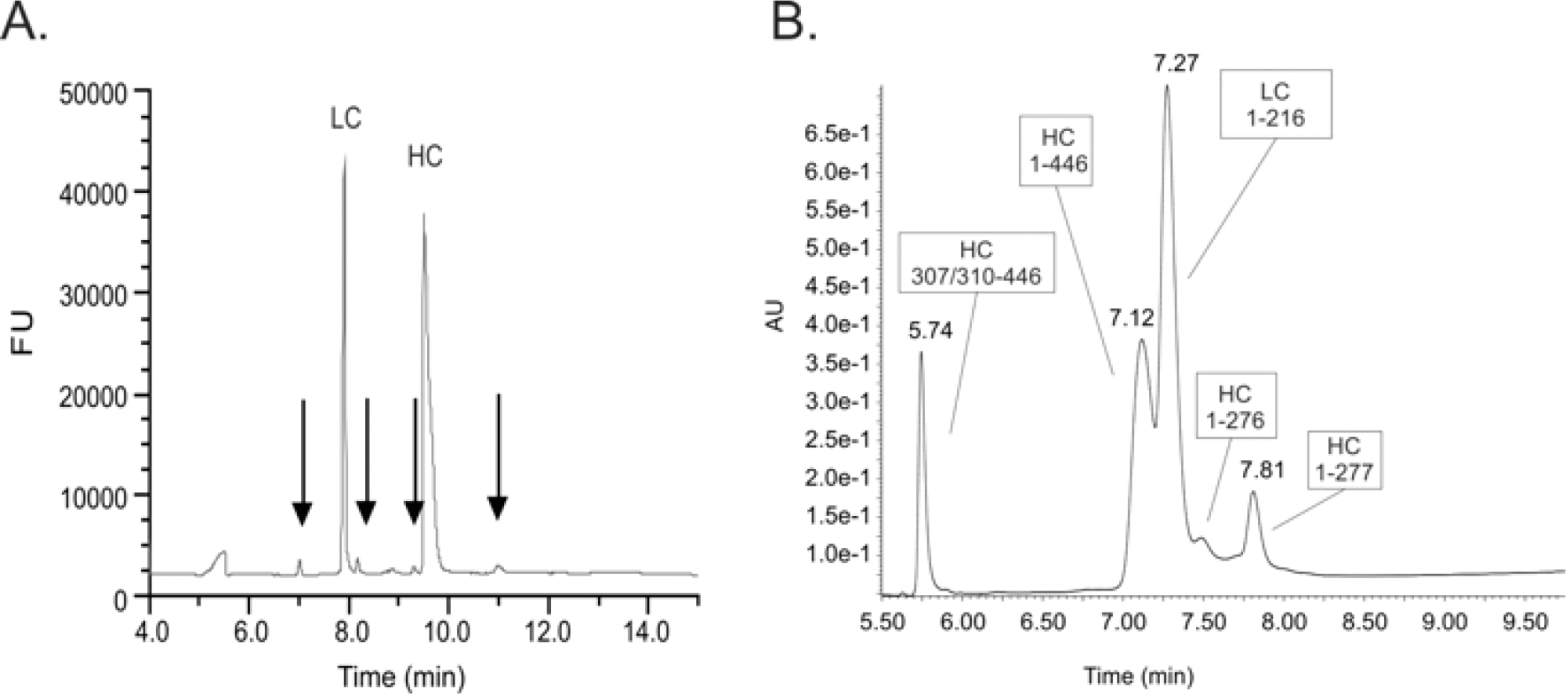
Characterization of fragment species A. Reduced CGE showing fragmentation of molecules (indicated with arrows) observed from a batch of mAb1. B. Reduced LC chromatogram (detected by MS) showing analysis of the sample exhibiting increased fragmentation.

**Figure 6:**
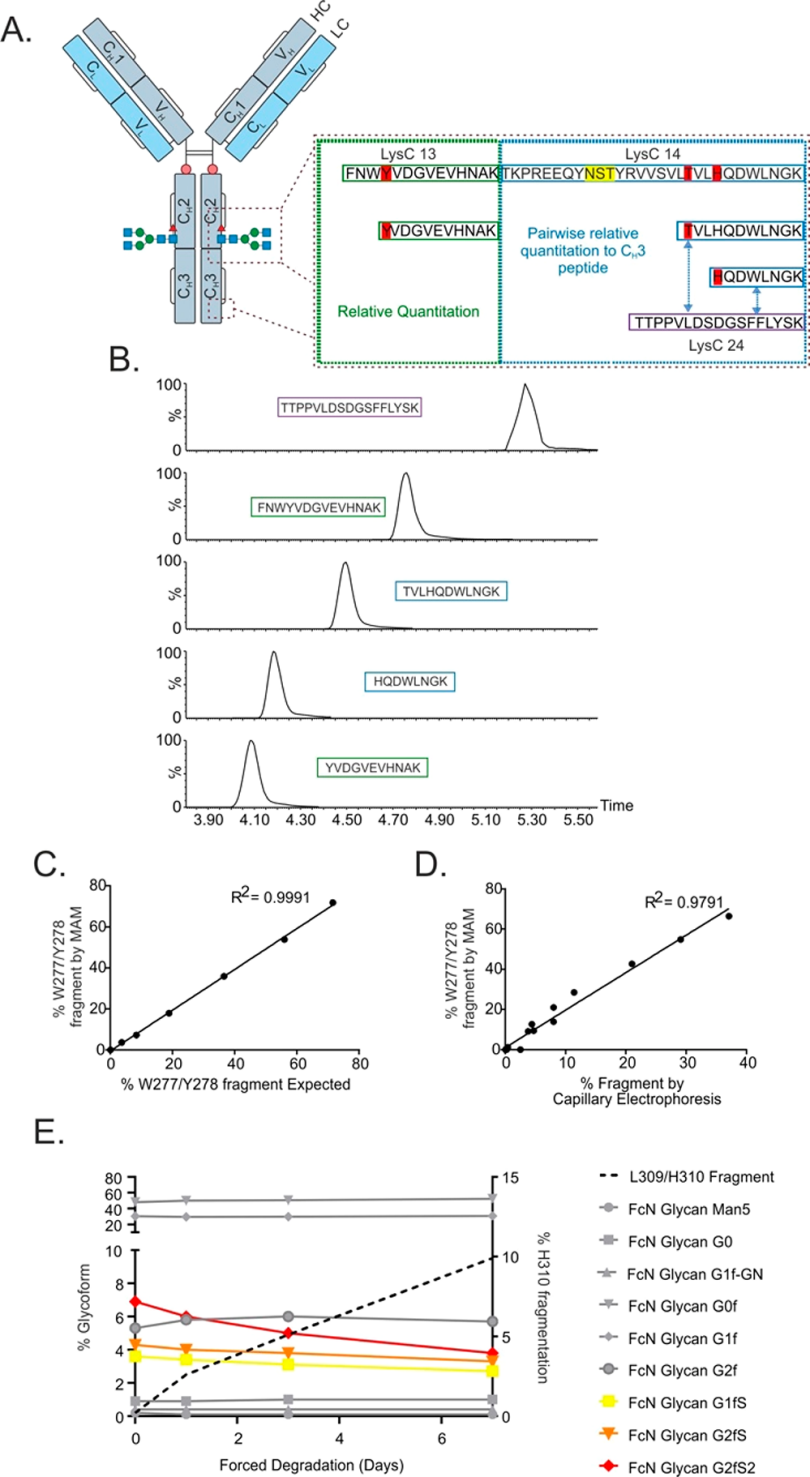
Transitions for identified common fragments, comparison to CGE, and MAM analysis of degraded samples. A. Schematic of three polypeptide fragments (W277/Y278, L306/T307, and L309/H310) monitored by the MAM method. The figure illustrates peptides detected to determine the relative fragmentation (in %), either through comparison with the unfragmented peptide or where multiple fragmentation sites occur, to a surrogate peptide conserved in the C_H_3 region. B. Fragment species MRM transitions labeled with their corresponding peptide. The MAM method utilizes these transitions to measure the relative level of peptide fragmentation at three sites where this is known to occur. C. Fragment species calibration curve for fragment 1 (W277/Y278) plotting expected vs measured percentage of polypeptide fragmentation. Material fragmented (through exposure to conditions known to cause fragmentation) was titrated into material which was not intentionally fragmented and measured by the MAM method. D. Comparison of the MAM method to the traditional capillary electrophoresis method. E. Monitoring site-specific fragmentation under force degradation conditions suggest sialylated glycopeptides (red/yellow/orange) fragment faster than peptides containing neutral glycoforms (gray).

Fragmentation of an antibody during manufacture and storage can occur by enzymatic (due to the presence of host cell impurities) or nonenzymatic-mediated reactions. Nonenzymatic fragmentation has been reported to be catalyzed by interaction with copper and iron; is highly pH-dependent; and is typically observed around the hinge region, domain–domain interface, and the complementary-determining region.^35,36^ Fragmentation of a mAb, however common, is usually not a major concern due to the drug product being highly purified and formulated in conditions which protect from fragmentation (or other degradation pathways). Interestingly, characterization of the C239i mAb intermediates by reduced capillary gel electrophoresis (CGE) revealed a unique and unexpected fragmentation pattern (Figure 5A). Intact reduced LC-MS (Figure 5B) identified four sites N276/W277, W277/Y278, L306/T307, and L309/H310 (the latter three are referred to as fragment 1, 2, and 3 respectively) within the C_H_2 region where increased fragmentation occurs (Figure 6A).

Transitions reporting on the three fragmentation sites were added to the MAM method to be able to monitor them (Figure 6B). Quantitation of fragment species is reported as a percentage relative to the full, unfragmented, peptide (Figure 6A), this relative quantitation is summarized by eq 3:
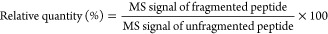
3

When this was not possible, for example, where multiple fragmentation sites occur across the same peptide, each fragmented peptide was quantified relative to a “standard” IgG1 peptide conserved within the C_H_3 region of all molecules analyzed, eq 4:
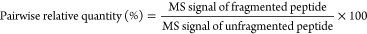
4

A sample enriched in fragmented mAb intermediate was titrated with unfragmented material to create a calibration curve. Comparison of the measured and expected level of fragment using the MAM method demonstrated a linear correlation for all 3 fragments (Figure 6C illustrates this for Fragment 1 (W277/Y278); Figure S7 illustrates correlations for Fragments 2 and 3 (L306/T307 and L309/H310, respectively)) had an R^2^ value of > 0.98. Validation of the MAM method for monitoring fragmentation was achieved by comparing results from this method to results generated from capillary gel electrophoresis, a traditional and orthogonal technology for quantifying fragmentation. The data were in broad agreement, whereby an increase in low molecular weight species (or the loss of a heavy or light chain) positively correlated (R^2^ = 0.98, Figure 6D) with the increase in fragmentation monitored by MAM. Tight correlation in the absolute level of fragmentation is not anticipated given the inherent differences in the measurement: capillary gel electrophoresis typically reports fragments as the proportion of total signal migrating earlier than heavy or light chains and does not accommodate for multiple fragmentation events or that chains are measured independently such that complete fragmentation of one chain (e.g., heavy chain) will not result in a complete loss of monomer purity since the other chain (e.g., light chain) will remain intact; conversely, MAM reports multiple fragments for a single molecule, measuring the extent of fragmentation at each site, and if combined could lead to double counting fragmentation of a given molecule.

Confident in the ability of the MAM method to accurately quantify fragmentation, we deployed it to characterize the rate of fragmentation in conditions we suspected promote fragmentation. Interestingly, in addition to an increase in fragmentation, we also observe a concomitant decrease in the signal derived from sialylated glycopeptides, suggesting that the rate of fragmentation at this site might be dependent upon the nature of the carbohydrate moiety of the molecule (Figure 6E).

## Discussion

A wide array of mass spectrometry technologies ranging from single quadrupoles to Fourier-transform ion cyclotron resonance (FTICR) can be employed to measure chromatographically separated peptides, each possessing advantages and shortcomings if compared.^37^ Here, we chose to use a triple quadrupole mass spectrometer and developed a multiple reaction monitoring method for monitoring C239i pCQAs since it satisfied our key method requirements: a linear response across a wide dynamic range (several orders of magnitude), acceptable sensitivity and selectivity (to 1% of each variant), good robustness (can routinely run ∼96 samples in a single sequence), and capable of fast and automated data acquisition and analysis. Traditional methods such as electrophoresis or chromatography do exist to monitor each quality attribute but were limited to a single method per quality attribute. Importantly, this approach allowed related and unrelated quality attributes to be analyzed within a single, quick analysis with the potential to further multiplex the method if required.

Samples (drug substance or in-process preparations of C239i mAbs) were digested to peptides using a nonreduced peptide mapping protocol. To avoid new disulfide bonds forming during sample preparation and analysis, free thiols were capped with *N*-ethylmaleimide (NEM), and disulfide bond (DSB) scrambling was minimized by digesting at pH 7 with the specific endopeptidase, *Lys-C*.^38^ A short (12 min) reversed phase gradient was developed to provide chromatographic separation of analytes with similar mass to charge ratios that would otherwise be difficult to discriminate by their mass to charge ratio alone (< 2 *m*/*z*). An abbreviated liquid chromatography gradient also facilitated automated sample injection, online sample desalting, and rapid column cleaning and re-equilibration. The final method was capable of confidently quantifying (relatively) variants of four quality attributes: the thiol state of the engineered cysteine, Fc glycoforms, partial reduction of interchain disulfide bonds, and polypeptide fragmentation, and was applied to support various stages of drug development. A detailed description of the method is provided in the Materials and Methods section.

Previously, we have reported the presence of unexpected thiol states of the C239i which exhibit differences in stability, structure, and dynamics to one another and wildtype IgG1.^26^ Intact mass spectrometry initially determined differences in the thiol states of C239i, but subsequent analysis showed that peptide level analyses were required to achieve the specificity necessary to accurately measure each. Standard peptide mapping protocols for mAbs can be lengthy (several hours of preparation, and LC-MS analysis time in the range of 70^39^ to 110^40^ mins per sample); therefore, a high-throughput targeted method was developed to be able to rapidly analyze many samples.

Inspiration for applying high throughput targeted mass spectrometry to multiplex the analysis of thiol states with other quality attributes was taken from similar approaches seen in both targeted bioanalysis,^41,42^ targeted proteomics,^37,43−45^ and targeted analysis of a single quality attribute.^46^ This approach extends work recently reported by others in the field of biotherapeutics who have shown the effectiveness of multiattribute monitoring methods in a QC environment,^9^ analyzing samples directly from a bioreactor,^12^ supporting bioprocess development in a controlled environment,^16^ and for specific monitoring of mAb N-glycosylation using intact mass MS.^14^

To measure thiol modifications, the samples needed to be digested under nonreducing conditions. Several of the resulting peptide analytes exhibited similar mass to charge ratios that required chromatographic separation to avoid coselection during quadrupole isolation. When exploring the ability of the method to provide a linear response for each thiol state, a signal bias was observed due to variation in the ionization propensity of each thiol state. This ionization bias was adjusted for by developing a correction factor which, when applied, provided linear correlation between expected and observed thiol variant values. Application of the method to support process development revealed that the initially unexpected thiol variant (iDSB) was observed consistently throughout the bioprocess, and that reduced states were observed only after harvest and protein A purification—a phenomenon believed to be caused by the presence of certain enzymes (released when cells are lysed) which disrupt mAb disulfide bonds.^33^ Thioredoxin and glutathione systems, being identified as enzymatic pathways, are most likely responsible for generating a reducing environment.^47^ Deeper characterization to explore the conditions under which iDSB forms was enabled by the high throughput nature of the analysis. We observed that iDSB variant forms spontaneously when there are free thiols present, and the rate of which is increased when the temperature is elevated to 37 °C and further still at 50 °C (Figure S5), suggesting greater dynamics increases the frequency of opposing inserted cysteines coming in close proximity to one another. Rapid formation of iDSB within 24 h at 37 °C after deglycosylation further supports the hypothesis that a steric constraint must be overcome to form the iDSB and is consistent with previous findings (Figure S6).^26^ Why the level of iDSB remains relatively constant (ca. ∼ 20%) during the fed-batch process remains unclear.

Higher than expected levels of sialylated glycans were observed during early development of the C239i constructs by the traditional 2-AB labeled, released glycan method. Transitions corresponding to these and other observed glycoforms were added to the MAM method, and studies were performed to bridge this approach to the conventional method. In-source fragmentation was minimized by optimizing source parameters through a design of experiments approach, extending work by Barton and co-workers who used a one factor at a time approach.^48^ Here, we generate a well-defined model of the parameter space, allowing the interdependencies of source parameters to be assessed and conditions to be determined that minimize in-source fragmentation while maintaining sensitivity. Differential ionization was addressed by determining normalization factors to correct for bias in signals owing solely to the nature of glycoform. Together, these optimizations allowed the successful quantitation of glycoforms by mass spectrometry and good correlation between the released-glycan HILIC and MAM methods. When applied, the method was capable of monitoring glycoforms at a throughput that was previously unachievable and demonstrated consistency of glycoforms within C239i constructs across different process stages, but notable differences in glycoforms between C239i and wildtype IgG1 constructs. Further work is required to elucidate the reason for increased sialylation in C239i antibodies with respect to wildtype IgG1; one possibility is that the increased dynamics of C239i molecules containing an additional disulfide bond help to accommodate larger, sialylated glycans.^45^ Methods that can concurrently measure these two attributes in individual molecules would help in substantiating this hypothesis.

Given the importance of the disulfide bond being maintained for structure/function, the relative novelty of the C239i construct whose format involves an unnatural sequence modification in close proximity to the hinge DSB, and the regulatory requirement to release antibody intermediate as a drug substance, transitions were added to the method which measured reduction in interchain disulfide bonds. The ionization bias on each reduced and nonreduced peptide was investigated, and significant bias was observed. To address this, a correction factor was determined and applied for both Kappa and Lambda chains alike. With these factors applied, the measurement for disulfide bond integrity was shown to be linear when compared to both expected values, and to the orthogonal, conventional assessment of antibody reduction by nonreduced capillary gel electrophoresis. When applied to characterize process samples, the method successfully identified C239i lots with reduced disulfide bond integrity.

Polypeptide fragmentation is a CQA which is conventionally mitigated by optimizing process steps, formulation, and storage conditions. Interestingly, fragmentation of the C239i construct was uncharacteristically high for an IgG1, and further investigation revealed fragmentation occurred at four sites within the C_H_2 region of the molecule that have not been previously described in the literature. Transitions to measure three of these were added to the MAM method to enable relative quantitation of these fragment species by either comparing them to the corresponding unfragmented peptide or to a “standard” peptide located in the C_H_3 region of the molecule, and good linearity and correlation to orthogonal methodologies were achieved. Studies (using the MAM method to measure fragmentation) were conducted which revealed fragmentation could be reduced by increasing the pH, initiating a process change that resulted in higher mAb intermediate purity. The unique placement of the fragmentation sites upon the N-linked glycopeptide allowed these to attributes to be evaluated concurrently and the generation of data that suggest molecules carrying sialylated glycoforms may fragment faster than those decorated with glycoforms typical of wildtype mAbs (G0f, G1f, G2f). To substantiate this observation requires further systematic characterization.

## Conclusion

The MAM method described here has been successfully applied to support preclinical drug development activities of the C239i antibody intermediate and understand the effect of upstream, downstream, and formulation/storage conditions on product quality. In addition to directing process development, we applied the MAM method to support product characterization: specifically, to explore the glycoform-dependent nature of C239i fragmentation, and the conditions required that favor iDSB formation (Figures S5 and S6). Previously, we have used this MAM method to measure thiol states of C239i throughout the conjugation process and revealed that iDSB is a likely contributor to under achieving the anticipated DAR.^26^

While the strengths of this approach are clear, and the work presented here demonstrates the utility of targeted mass spectrometry not just for engineered antibodies but for biologics in general, targeted acquisition methodologies are not without their limitations. Principally, this approach is limited in its ability to only acquire data for attributes that are known a priori; the method is modular and evolves with time whereas untargeted analyses allow for retrospective analysis of the full MS1 scan and a selection of MS2 data (data dependent acquisition, DDA) or full MS1 scan and all ensuing fragment data (data independent acquisition, DIA).

MAM methods which employ a more global approach to MS data acquisition are becoming popular in the field of biotherapeutic characterization,^18^ where peptide mapping methodology is being modified or streamlined^11^ to monitor critical attributes while providing the option for retrospective searching of data if new attributes are discovered.

There is yet no panacea for this methodology as shortfalls such as low throughput, long method times, demand for complex data interpretation, and the need for expensive high-resolution instruments limit their widespread application. Additional steps have already been taken to further streamline analyses such as subverting the traditional LC-MS approach (that often requires long gradients to resolve critical modifications) to include orthogonal separations in tandem, such as ion-mobility separation where ions are separated within the mass spectrometer according to their collisional cross section value,^49^ thus reducing the need for lengthy LC separations. Alternatively, methods that utilize higher resolution mass analyzers with ever faster duty cycles, or data-independent acquisition approaches that remove the need to isolate precursors prior to fragmentation (MS^E^^50^ or SWATH^51^) all hold potential to increase the throughput of untargeted analyses. Looking further ahead, improvement of multi-attribute monitoring approaches that start at the protein level (without requiring digestion) such as subunit^13^ and top-down^52^ analyses may ultimately provide a path to greater information content at a higher throughput that could eventually supersede the need for peptide level analysis altogether.

## Supporting Information Available

Additional experimental design, data, and analyses (PDF)
