# Supplementary material for: Multi-Attribute Monitoring Method for Process Development of Engineered Antibody for Site-Specific Conjugation
Source: J Am Soc Mass Spectrom. 2023 Jun 2;34(7):1330–41. doi: 10.1021/jasms.3c00037 (PMC10326913; doi:10.1021/jasms.3c00037)
Supplement: Supplementary file 1 — js3c00037_si_001.pdf [file js3c00037_si_001.pdf]

Multi Attribute Monitoring Method for Process Development of Engineered Antibody for Site-Specific Conjugation

Alistair R. Hines[a]*, Matthew Edgeworth[a], Paul W. A. Devine[a], Samuel Shepherd[a], Nicholas Chatterton[b], Claire Turner[c], Kathryn S. Lilley[d], Xiaoyu Chen[e], and Nicholas J. Bond[a]*.

[a]Analytical Sciences, Biopharmaceutical Development, R&D, AstraZeneca, Cambridge, CB2 0AA, UK

[b]The Open University, Walton Hall, Milton Keynes, MK7 6AA, UK

[c]College of Health, Medicine & Life Sciences, Brunel University London, Middlesex, UB8 3PH, UK

[d]Cambridge Centre for Proteomics, Department of Biochemistry, University of Cambridge, Cambridge, CB2 1QR, UK

[e]Analytical Sciences, Biopharmaceutical Development, R&D, AstraZeneca, Gaithersburg, MD 20878, USA

All work completed in Cambridge, Cambridgeshire, UK.

Corresponding Author

*Alistair R. Hines, alistair.hines@astrazeneca.com

*Nicholas J. Bond, nick.bond@astrazeneca.com

Supplementary material

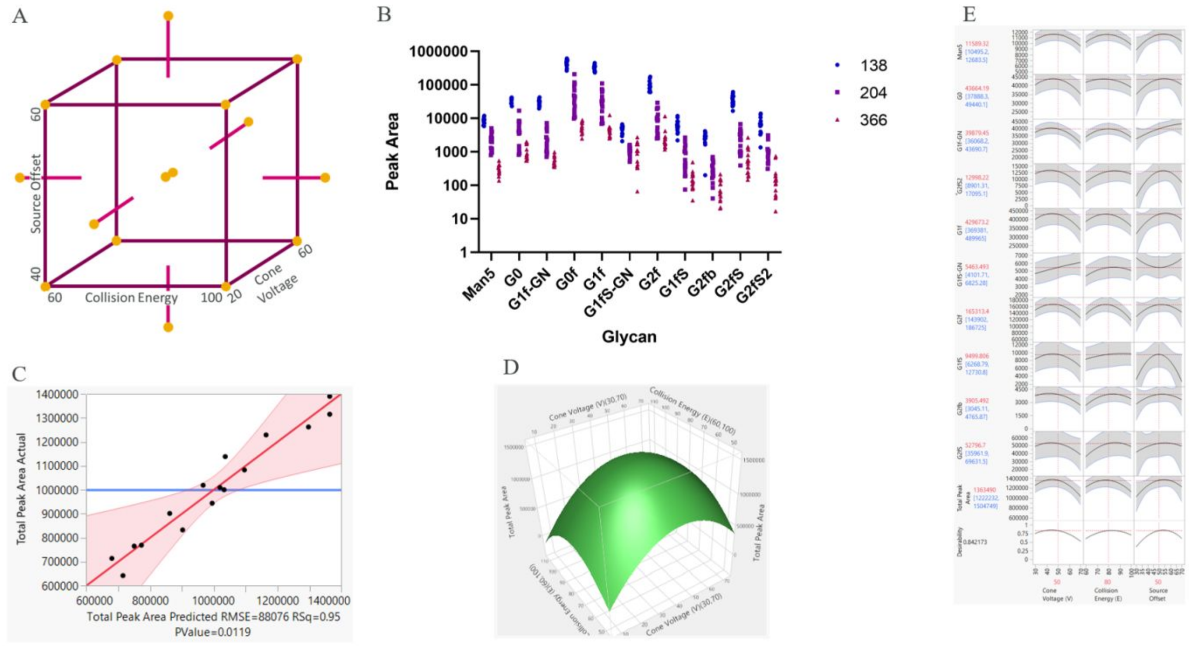

Figure S. 1: A. DoE schematic of parameter space modelled for impact on glycopeptide signal (source offset 40-60 V, collision energy 60-100 V, and cone voltage 20-60 V).

B. Absolute response values across all glycans measured for the three oxonium ions, showing without exception that *m/z* 138 gives the highest response values.

C. Actual vs predicted plot for the DoE demonstrates good model fit as predicted values for peak area correlate strongly with observed values ($R^2 = 0.95$).

D. Surface profiler highlighting the non-linear impact of changes to cone voltage and collision energy on the signal (total peak area) of all glycopeptides.

E. Prediction profiler showing impact of variable changes on individual and total glycopeptide signal. Use of the maximised desirability function predicts optimal conditions within the ranges for cone voltage 40-50 V, source offset 50-60 V, and collision energy 70-80 V.

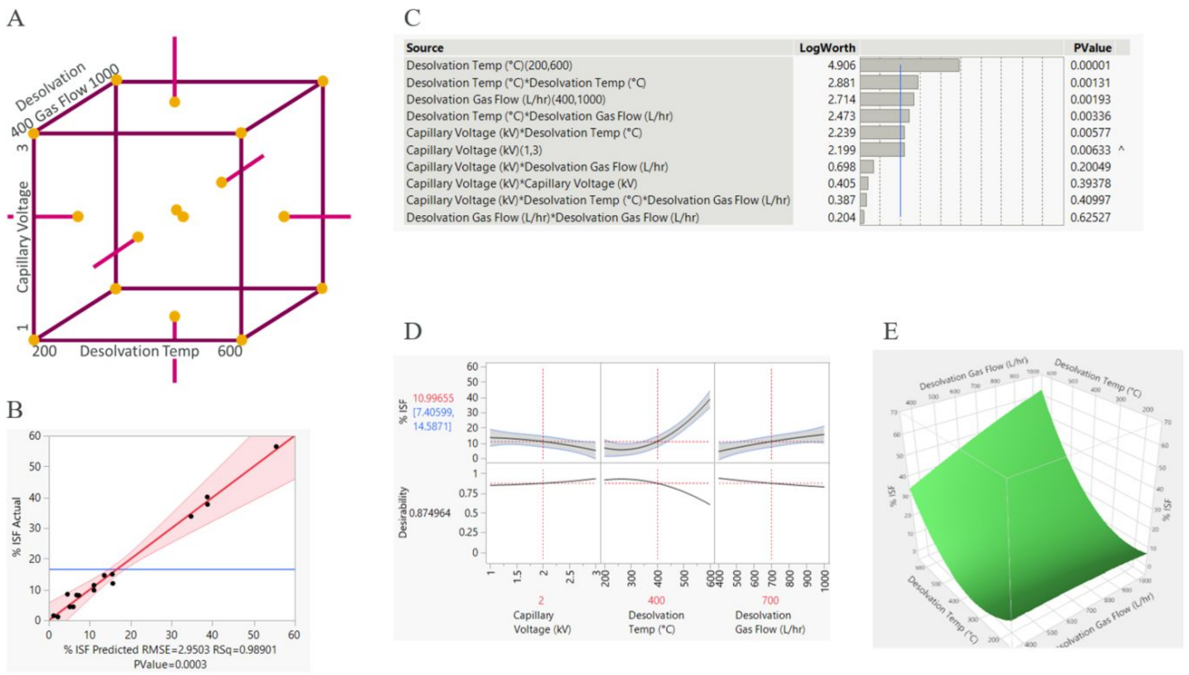

Figure S. 2: **A.** DoE schematic of parameter space modelled for impact on in-source fragmentation (desolvation gas flow 400-1000 L/hour, capillary voltage 1-3 kV, desolvation temperature 200-600 °C).

B. Actual vs predicted plot for the DoE demonstrates good model fit as predicted values for in source fragmentation correlate strongly with observed values ($R^2 = 0.99$).

C. Effect summary report detailing the impact of each parameter on in source fragmentation with statistically significance (P value).

D. Prediction profiler showing impact of variable changes on in source fragmentation. Use of the maximised desirability function predicts optimal conditions as a desolvation gas flow of 400 L/hour, capillary voltage of 3 kV, and desolvation temperature of 350-450 °C, whilst not significantly impacting glycopeptide signal (data not shown).

E. Surface profiler highlighting the impact of changes to desolvation temperature and gas flow on the level of in source fragmentation.

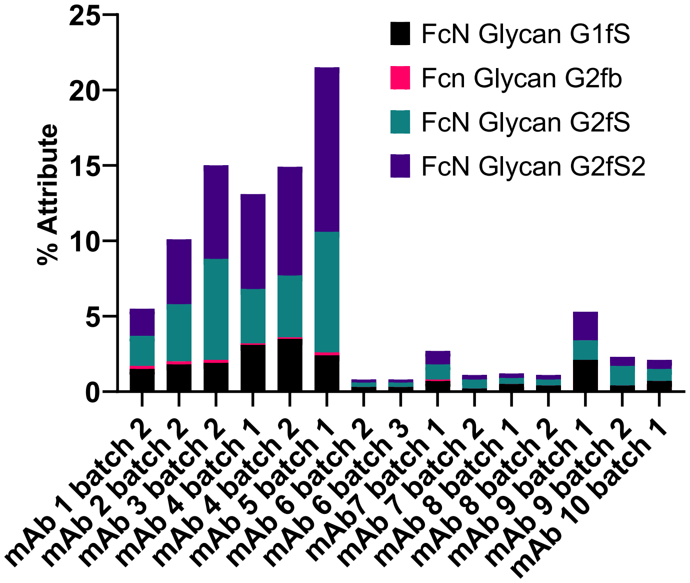

Figure S. 3: Comparison of sialylated glycans observed for both C239i antibody intermediates (mAb 1-5, left) and standard mAb reference materials (mAb 6-10, right).

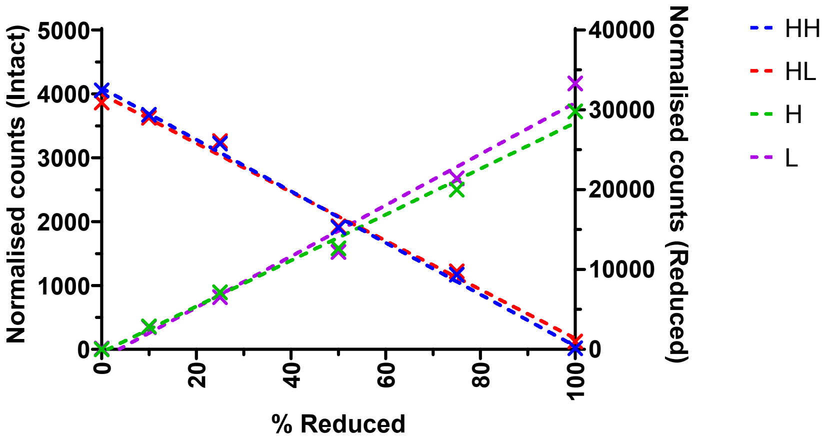

Figure S. 4: Shows normalised counts for the various Kappa chain peptides with data points showing that, as the % reduction increases (from spiking fully reduced material into starting material at known ratios), the signal for the reduced peptides monitored (H,L) increase while the signal for disulphide-bonded peptides decreases.

The legend gives shorthand for the proteotypic peptide measured:

- HH – 2 disulphide bonded heavy chains. Intensity of signal starts high and decreases as the amount of reduced species increases, indicating the protein (only the peptide containing two s-s bonds is measured) is being reduced.
- HL – a disulphide bonded pair of heavy/light chains. Intensity of signal starts high and decreases as the protein is reduced, again, showing this pairing being split.
- H/L – free heavy/light chain. For both species we see this signal increase from 0 (showing no detected free chain at no imposed reduction) linearly to highest values being at 100% reduction

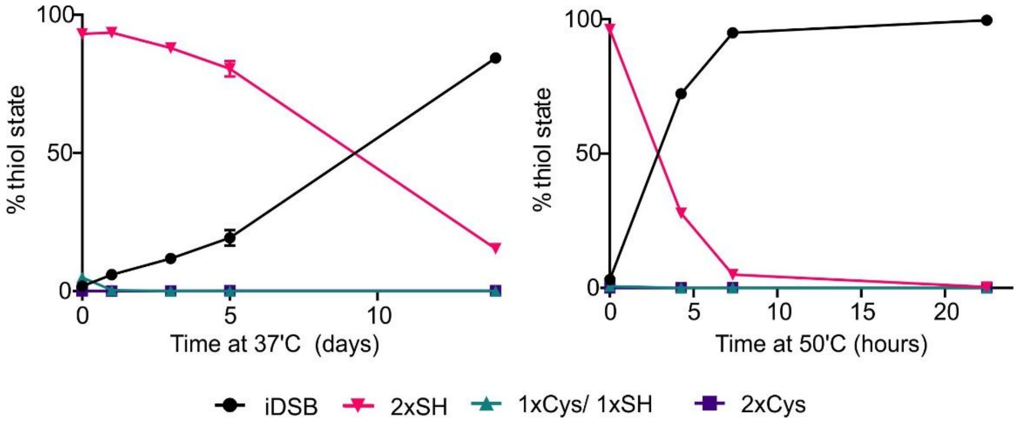

Figure S. 5: Effect of heat on the thiol state evolution over time starting from 2xSH enriched material: A. at 37 ˚C, B at 50 ˚C.

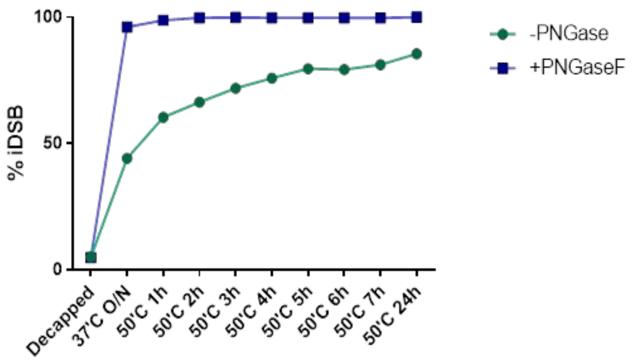

Figure S. 6: Effect of glycosylation on % iDSB when incubating with L-Cysteine over time at different temperatures, showing that the presence of glycans inhibit the formation of iDSB.

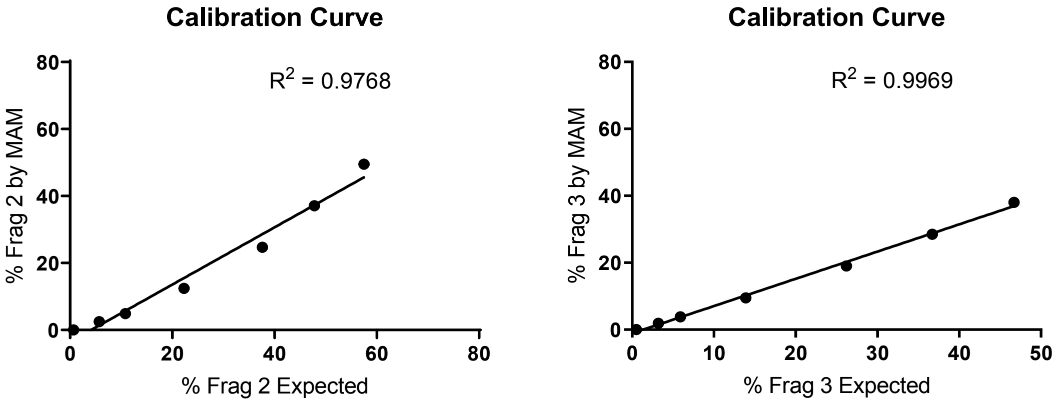

Figure S. 7: Fragment species calibration curve for fragment 2 and 3 (T307 and H310 respectively) plotting expected vs measured percentage of polypeptide fragmentation. Material fragmented (through exposure to conditions known to cause fragmentation) was titrated into material which was not intentionally fragmented and measured by the MAM method.

a)

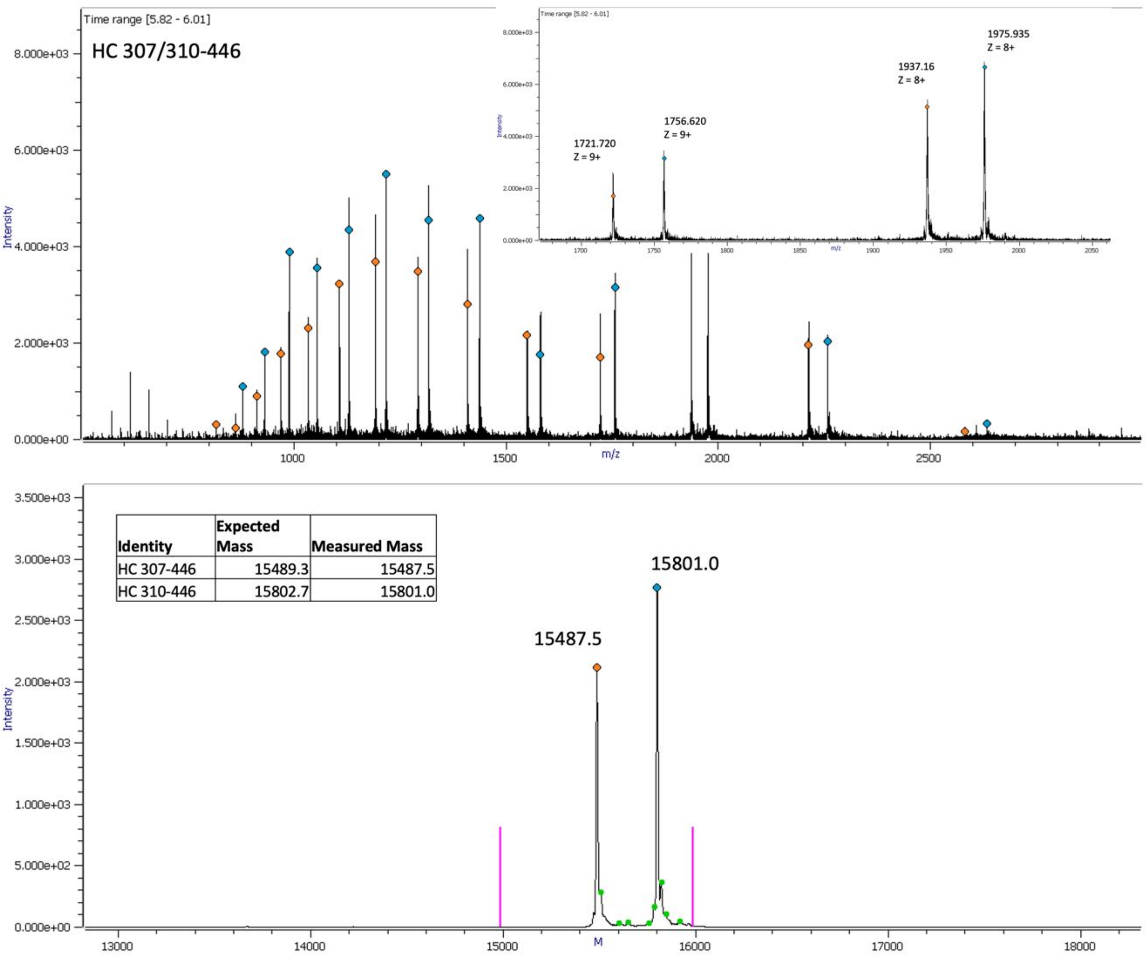

Identity	Expected Mass	Measured Mass
HC 307-446	15489.3	15487.5
HC 310-446	15802.7	15801.0

b)

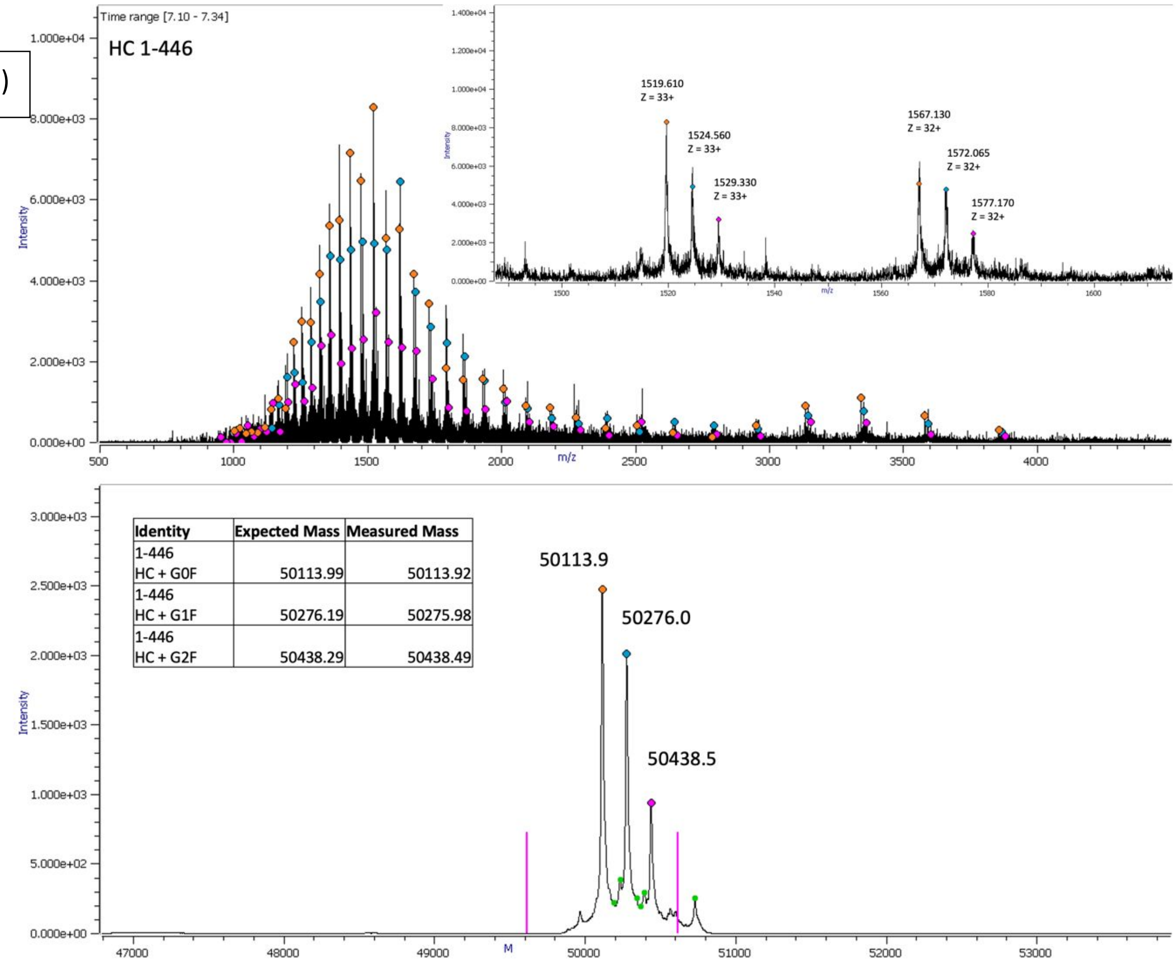

Identity	Expected Mass	Measured Mass
1-446 HC + G0F	50113.99	50113.92
1-446 HC + G1F	50276.19	50275.98
1-446 HC + G2F	50438.29	50438.49

c)

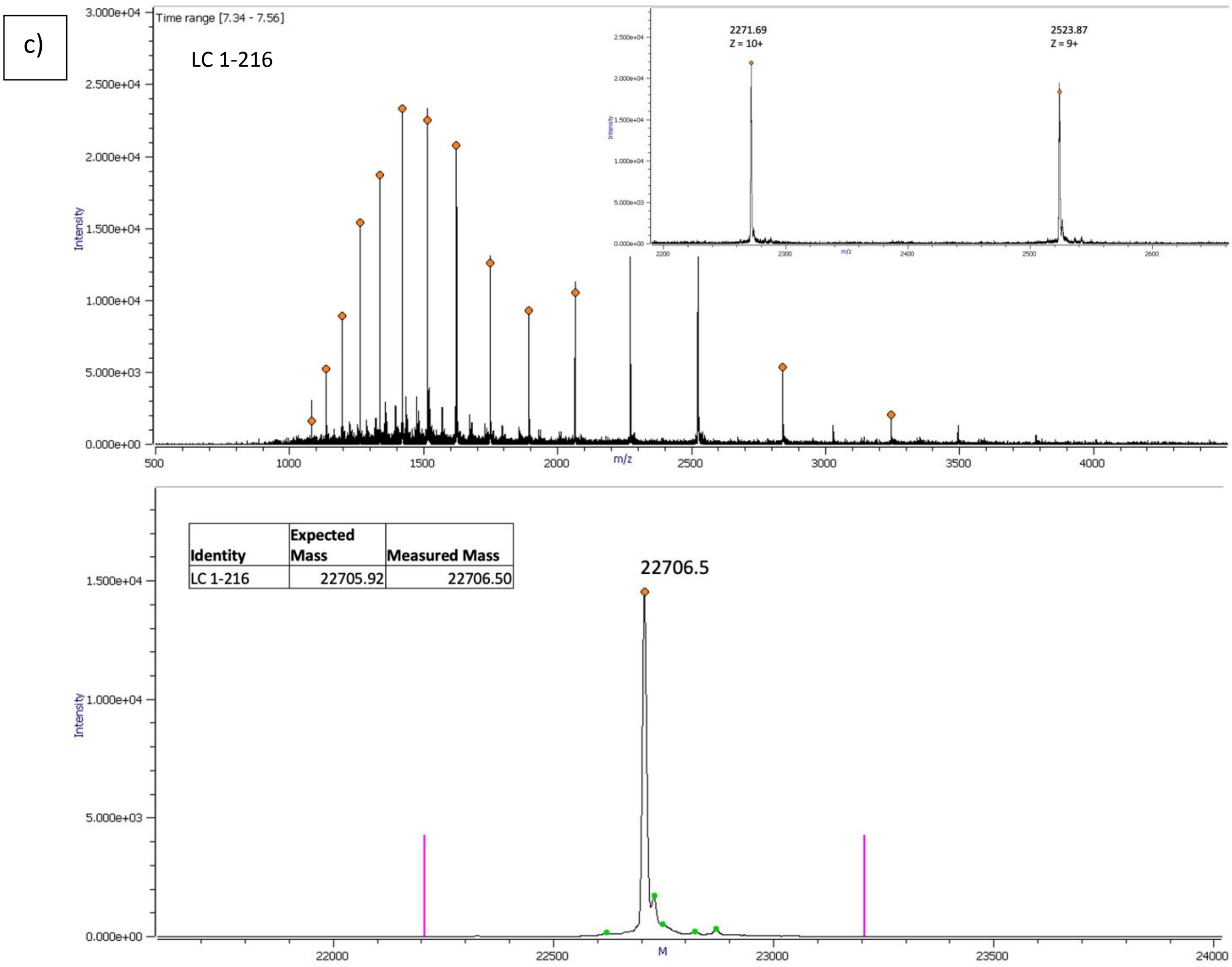
Time range [7.34 - 7.56]
LC 1-216
2271.69
Z = 10+
2523.87
Z = 9+
Intensity
m/z
Identity
Expected Mass
Measured Mass
LC 1-216
22705.92
22706.50
22706.5
M

d)

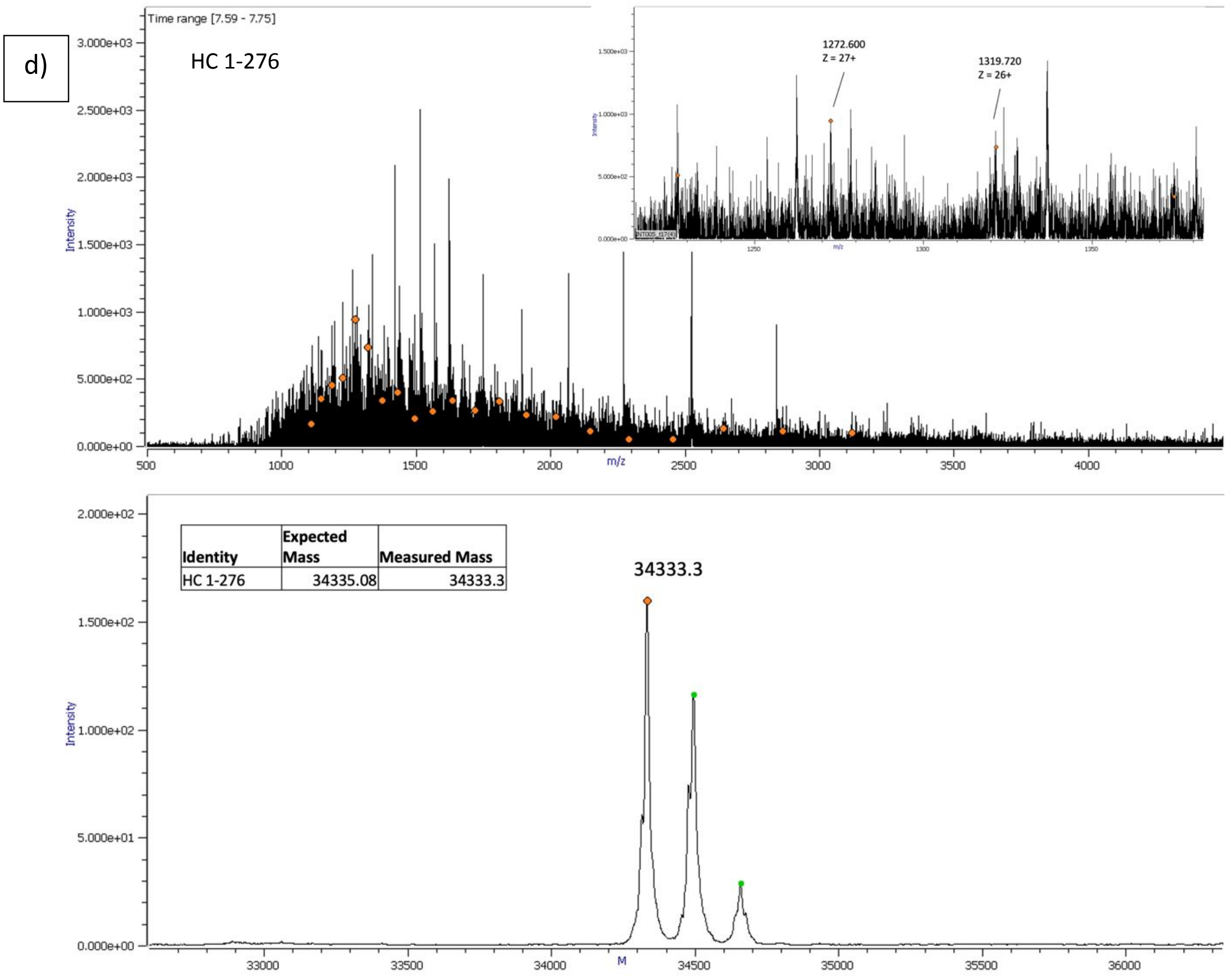

Identity	Expected Mass	Measured Mass
HC 1-276	34335.08	34333.3

e)

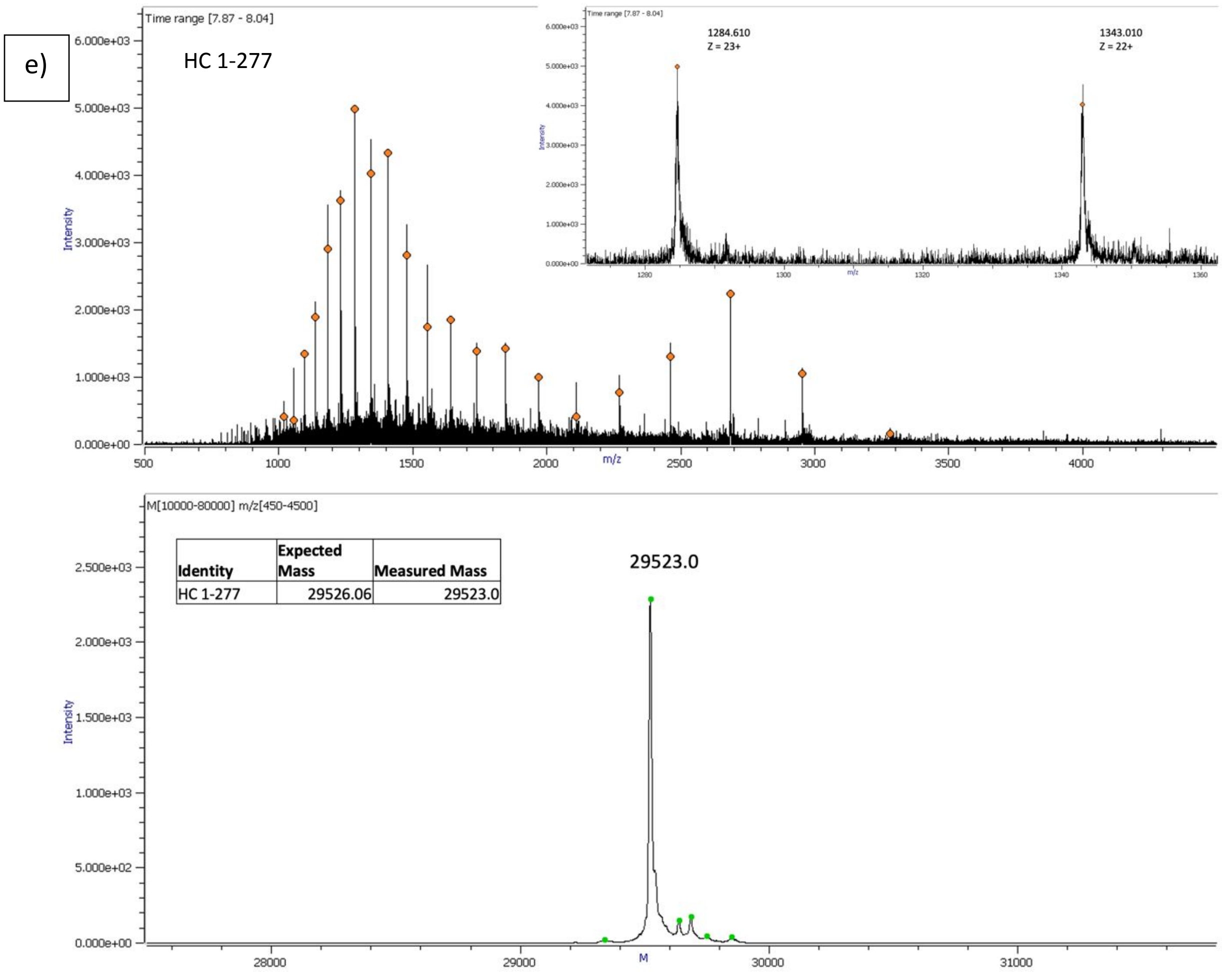

Figure S. 8: Raw *m/z* (top and insert) and deconvolved (bottom) MS data for the 5 peaks labelled in Figure 5B. a) HC 307/310-446; b) HC 1-446; c) LC 1-216; d) HC 1-276; e) HC 1-277.
